# Robustness of five different visual assessment methods for the evaluation of hindlimb lameness based on tubera coxarum movement in horses at the trot on a straight line

**DOI:** 10.1111/evj.13531

**Published:** 2021-12-13

**Authors:** Sandra D. Starke, Stephen A. May

**Affiliations:** ^1^ The Royal Veterinary College North Mymms Hatfield UK

**Keywords:** hindlimb lameness, horse, tubera coxarum

## Abstract

**Background:**

The evaluation of hindlimb lameness remains a major challenge in everyday clinical practice. In the absence of clear guidelines, veterinarians use different visual assessment methods for this task whose robustness is unknown.

**Objectives:**

Determination of the robustness of five visual hindlimb lameness assessment methods based on the comparison of left and right tuber coxae movement.

**Study design:**

Validated mathematical hindlimb lameness model based on experimental data from the literature.

**Methods:**

Vertical movement of left (LTC) and right (RTC) tuber coxae was simulated for the range of common hindlimb lameness movement patterns that horses present within practice. Lameness severity ranged from sound to moderately lame (0% to 60% motion asymmetry). The scenarios of a pelvis held tilted and asymmetrical pelvic roll were included to reflect possible adaptations in pelvic rotation. Across all conditions, the outcomes for five different visual assessment methods based on comparative tubera coxarum movement were quantified, including hip hike, ‐drop and range of motion. The robustness of each assessment method was established through comparison to sacrum‐based overall motion asymmetry as the ground truth.

**Results:**

Tubera coxarum‐based lameness assessment was highly sensitive to all the unique lameness patterns and changes in pelvic rotation which a lame horse may adopt. None of the five visual lameness assessment methods was 100% robust across all conditions tested. For everyday clinical practice, comparing the upward movement amplitude of the RTC before right hind foot contact and of the LTC before left hind foot contact (Hip_hike_diff) would be the most robust single tubera coxarum‐based visual assessment method.

**Main limitations:**

In the absence of published data regarding the frequency of different movement patterns and hip rotation adaptations in clinical practice, this study cannot indicate the proportion of assessments that would be incorrect for a given visual assessment method.

**Conclusions:**

Using a single tubera coxarum‐based visual hindlimb lameness assessment method may lead to incorrect clinical judgement. Therefore, using multiple assessment methods would be beneficial to substantiate impressions.

## 1 INTRODUCTION

Compared to forelimb lameness, hindlimb lameness is considered more difficult to assess visually,^1^, ^2^, ^3^ is seen less frequently in equine practice^4^ and shows very poor classification reliability.^5^, ^6^, ^7^, ^8^ Part of this issue may arise from differences in the approach and assessment methods to detect lameness,^9^ where variation in and sometimes contradictory descriptions of the signs of hindlimb lameness have long been highlighted.^2^, ^9^, ^10^ Further, during a trot, the pelvis shows a smaller overall vertical range of movement compared to the head,^11^ potentially making motion asymmetry harder to determine.

Hindlimb lameness can be determined by evaluating the movement of either the sacrum or comparing that of both tubera coxarum.^2^ The visual (and quantitative) assessment of sacrum movement (a)symmetry at the trot on a straight line is reasonably straightforward: a non‐lame horse presents with two symmetrical vertical sacrum excursions per stride, whereas a lame horse presents with increasing asymmetry between these two excursions. The assessment of the tubera coxarum during the lameness assessment is more complex: movement of the left and right tubera coxarum has to be compared since pelvic rotation and translation interact to produce an overall movement pattern that is by default asynchronous and asymmetrical between sides.^11^, ^12^, ^13^ Yet, despite this complexity, visual assessment of the tubera coxarum is often used in clinical practice for hindlimb lameness evaluation and is also commonly described in the clinical literature as one of the key indicators of hindlimb lameness.^1^, ^2^, ^4^

Currently, there is no standardised lameness assessment protocol with regards to what specifically clinicians should be looking for and how it should be weighted, paired with the lack of one standard lameness grading scale. Possible visual lameness assessment methods include — and may combine — upward movement amplitudes such as the “hip hike” or positional features such as the lowest position during the stride. Most or all of these assessment methods hold relevant information.^14^ However, different lame horses show different overall pelvis movement patterns,^15^, ^16^, ^17^ which impact the observable motion asymmetry of the horse. As a consequence of these interacting factors, a given visual assessment method might work well for one lameness pattern or horse but not for another. This constraint in the reliability of different visual assessment methods has to date not been systematically investigated and remains poorly understood.

Mathematical modelling provides an opportunity to explore the continuous, systematic behaviour of visual (and measurement‐based, respectively quantitative) hindlimb lameness assessment methods across a range of lameness severities and movement patterns where there are limited datasets. We previously described the relationship between the movement of the sacrum and tubera coxarum in the context of different lameness patterns.^13^ This previous work demonstrated that a geometrical model predicts experimental data describing pelvic movement recorded from 107 horses extremely well, ranging from horses being sound to moderately lame.^13^ The current work used this model to explore what we can expect from five visual hindlimb lameness assessment methods that compare left and right tubera coxarum across a range of conditions expected to be encountered in practice.

The aim of this study was to determine the robustness and limitations of five tubera coxarum‐based visual assessment methods for the evaluation of hindlimb lameness. We systematically investigated: (a) The ability of these comparative tubera coxarum‐based assessment methods to correctly classify horses presenting with different lameness adaptation patterns and (b) the robustness of these methods to perturbations in pelvic rotation. Based on prior clinical observations and a published model, we hypothesised that different visual assessment methods would incorrectly classify lameness for specific pelvic movement patterns and pelvic rotation adaptations. The findings are not only highly relevant to the understanding of visual gait assessment, but also to the interpretation of quantitative/objective gait analysis.

## 2 MATERIALS AND METHODS

### 2.1 Tubera coxarum‐based lameness assessment methods

**FIGURE 1:**
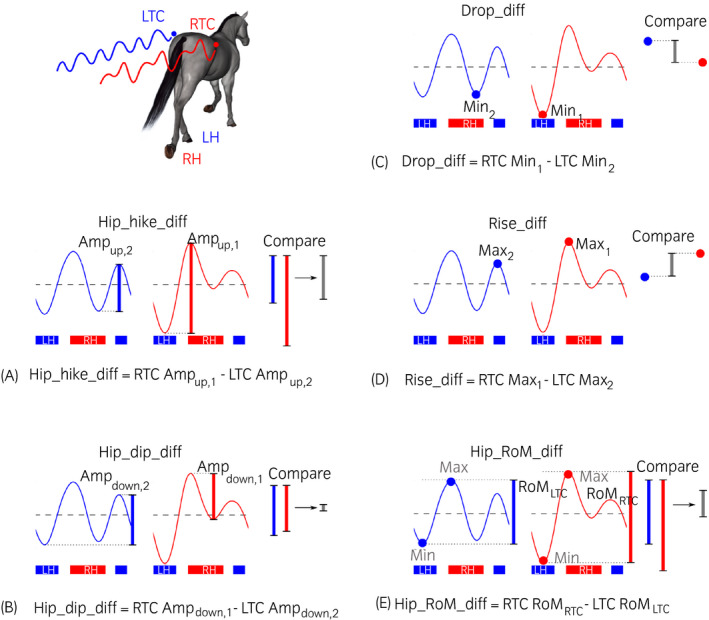
Five popular hindlimb lameness assessment methods and their calculation based on comparative vertical tubera coxarum displacement. Hip_hike_diff (A) and Hip_dip_diff (B) compare differences (diff) in amplitudes between left and right tuber coxae during specific timings of the stride. Drop_diff (C) and Rise_diff (D) compare differences in absolute positions between left and right tuber coxae during specific timings of the stride. Hip_RoM_diff (E) compares differences in the overall range of movement (RoM) between left and right tuber coxae irrespective of stride timings. Amp_down_, downward movement amplitude; Amp_up_, upward movement amplitude; LH, left hindlimb; LTC, left tuber coxae; Min, minimum and Max, maximum; RH, right hindlimb; RoM, range of movement; RTC, right tuber coxae. Stance phases are indicated as horizontal bars. For the definition of visual assessment methods, please refer to Table 1

The behaviour of five tubera coxarum‐based visual lameness assessment methods (Table 1) was quantified based on comparative left and right tubera coxarum movement during different parts of the trot stride cycle (Figure 1). This comprised three assessment methods based on relative movement of the left and right tuber coxae (Hip_hike_diff, Hip_dip_diff Hip_RoM_diff, RoM – Range of Movement) and two assessment methods based on the actual heights/positions of the left and right tuber coxae (Drop_diff, Rise_diff). All visual assessment methods are described in detail in Table 1.

**TABLE 1:** Five visual lameness assessment methods based on the comparison of left and right tuber coxae

Abbreviation	General assessment method	Explanation
Hip_hike_diff	Assessment of upward movement amplitudes with regard to relative distance travelled at specific stride timings.	An assessor looks for the tuber coxae of the lame limb to hike up before foot contact of that limb. Upward movement of the LTC and RTC is compared before foot contact of the respective limb (see also Figure 1a). Hip_hike_diff is, therefore, the difference between the upward movement amplitude of the RTC *before* RH foot contact and of the LTC *before* LH foot contact.
Hip_dip_diff	Assessment of downward movement amplitudes with regard to relative distance travelled at specific stride timings.	An assessor looks for the tuber coxae of the lame limb to drop more during the stance of that limb. Downward movement of the LTC and RTC is compared during the stance of the respective limb (see also Figure 1b). Hip_dip_diff is, therefore, the difference between the downward movement amplitude of the RTC *during* RH stance and of the LTC *during* LH stance.
Drop_diff	Assessment of actual heights of the landmarks in space at specific stride timings.	An assessor looks for the tuber coxae of the lame limb to reach the lowest absolute position of both tubera coxarum during the whole stride. The position of LTC and RTC is compared during the stance/end of the stance of the contralateral limb (see also Figure 1c). Drop_diff is, therefore, the difference between the minimum height of the RTC *during* LH stance/foot off and of the LTC *during* RH stance/foot off.
Rise_diff	Assessment of actual heights of the landmarks in space at specific stride timings.	An assessor looks for the tuber coxae of the lame limb to reach the highest absolute position of both tubera coxarum during the whole stride. The position of LTC and RTC is compared before foot contact of the respective limb (see also Figure 1d). Rise_diff is, therefore, the difference between the maximum height of the RTC *before* RH foot contact and of the LTC *before* LH foot contact.
Hip_RoM_diff	Assessment of range of movement with regard to relative distance travelled across the whole stride.	An assessor looks for the tuber coxae with the greatest overall movement range to declare the respective limb lame. Tuber coxae movement is observed across the whole stride cycle (see also Figure 1e). Hip_RoM_diff is, therefore, the difference between the whole movement range of RTC and LTC irrespective of stride timings.

Note

Five visual lameness assessment methods based on comparative tubera coxarum assessment were investigated in this study. These methods were deducted from descriptions in the literature^2^ and conversations with a range of veterinary experts (SD Starke, unpublished data). Please refer to Figure 1 for illustrations and calculations.

Abbreviations: LH, left hindlimb; LTC, left tuber coxae; RH, right hindlimb; RTC, right tuber coxae.

### 2.2 Mathematical hindlimb lameness model

**FIGURE 2:**
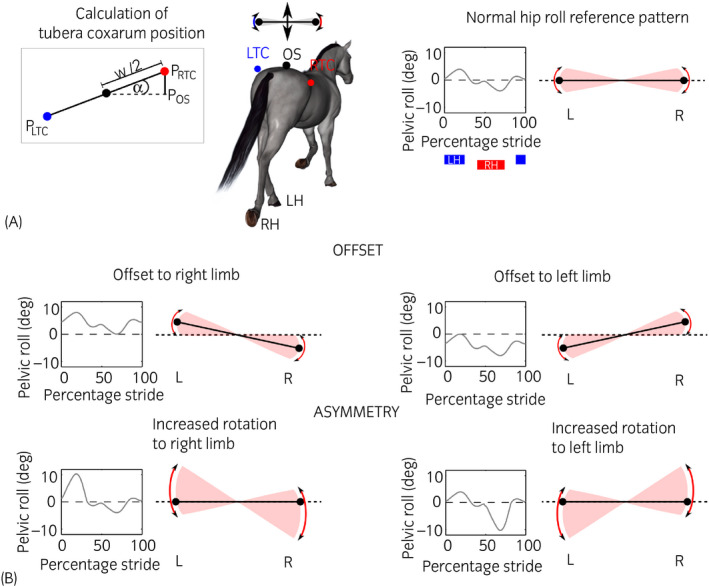
Summary of the geometrical hindlimb lameness model (A) and illustration of the performed perturbations to pelvic roll (B), introducing an offset (pelvis held tilted towards one side) and pelvic roll asymmetry

To examine the ability of the five visual lameness assessment methods to identify lameness correctly across a broad spectrum of potential lameness severities and movement patterns, a geometrical model of pelvis movement for sound to moderately hindlimb lame horses was created in Matlab 2018b (The MathWorks) as described and validated previously.^13^ This model assumed a rigid connection between the sacrum and tubera coxarum (Figure 2) and was previously shown to predict movement patterns across 107 lame horses with very good fit.^13^

**FIGURE 3:**
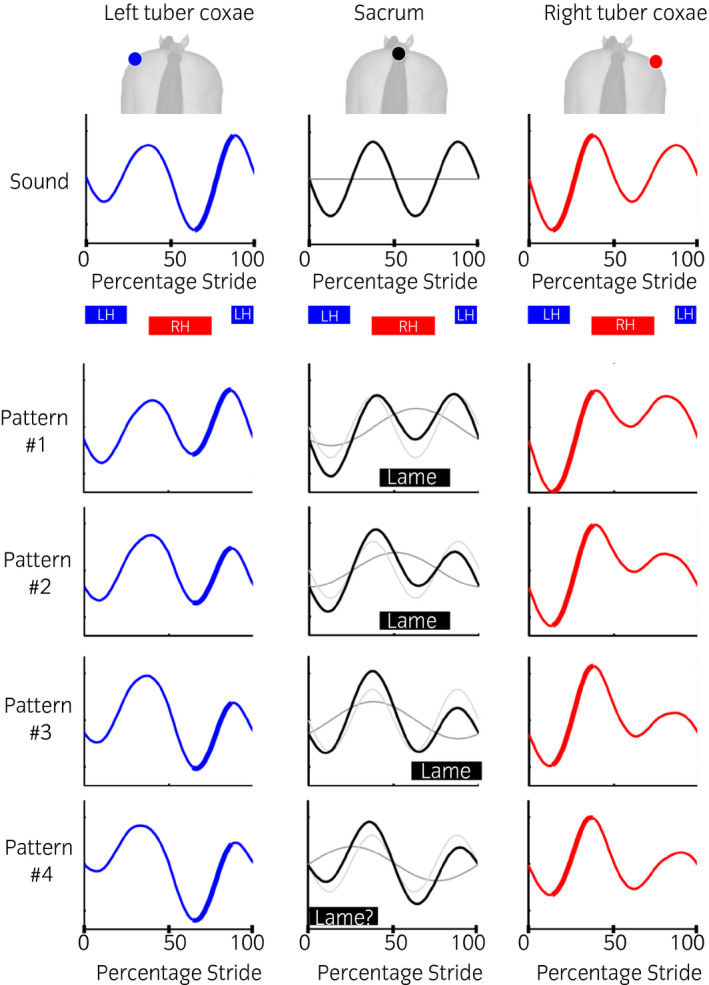
Four general vertical sacrum displacement patterns observed in hindlimb lameness (middle column, black), covering the boundaries across the full spectrum of possible patterns observed in clinical practice.^15^ Asymmetrical movement of the sacrum (black) is the result of a symmetrical (light grey, A2) and asymmetrical (dark grey, A1) movement component that are shifted in time relative to each other, defining each of the four patterns. The resulting tubera coxarum movement (blue – left tuber coxae, red – right tuber coxae) differs according to the movement pattern of the sacrum. Bold: amplitudes indicating the same movement during the stride cycle for each contralateral limb. Sound horse: no difference between the height of the two sarum maxima or minima. Pattern #1 – difference between the height of the sacrum minima only, pattern #2 – difference between the height of both sacrum maxima and minima (the second minimum is higher than the first one and the second maximum is lower than the first one), pattern #3 – difference between the height of the sacrum maxima only, pattern #4 – difference between the height of both, sacrum maxima and minima (the second minimum and maximum are both lower than the first ones); for this pattern, it is unclear how to interpret it with regard to the lame limb

In summary, the model generates vertical tubera coxarum displacement trajectories based on (a) an average pelvic rotation pattern trajectory (derived experimentally) and (b) computer‐generated sacral displacement trajectories for a continuum of lameness severities. Vertical displacement of the left (LTC) and right (RTC) tuber coxae (respectively, the vertical tubera coxarum position throughout the stride cycle (*P*_RTC, LTC_)) was calculated from these pelvic roll‐ and scrum displacement trajectories using trigonometry (Figure 3). The trigonometry is based on hip width (*w*, distance between both tubera coxarum based on an average of 0.55 m estimated from four Thoroughbred horses^13^), sacral position (*P*_OS_) and pelvic rotation (*α*) as:

$$P_{\text{RTC}}=P_{\text{OS}}‐\sin\left(\alpha \right)\frac{w}{2}\;\text{and}$$

$$P_{\text{LTC}}=P_{\text{OS}}+\sin\left(\alpha \right)\frac{w}{2}.$$

Key to deriving the tubera coxarum movement trajectories for subsequent calculation of the behaviour of the five visual lameness assessment methods were the computer‐generated sacral displacement trajectories. These sacrum trajectories defined a broad range of movement adaptations for hindlimb lame horses, backed by experimental data, from which tubera coxarum displacement can easily be deducted as a function of pelvic rotation. Sacral displacement trajectories for various lameness grades and patterns were generated by reversing a signal decomposition approach to lameness detection used for experimental data captured from live lame horses,^15^, ^16^ where a symmetrical (A2) and asymmetrical (A1) signal component were summed to reconstruct observable movement. Lameness severity was controlled through the ratio between the signal components A1 and A2, with overall motion asymmetry (MAS, see below) of the sacrum ranging from 0% to 60%. This overall motion asymmetry of the sacrum was used as the reference for a given simulated horse's lameness state (see data analysis for details). The vertical movement pattern of the sacrum was controlled through the phase shift (offset in time) between A1 and A2. In this study, a shift between the A1 and A2 signal for four distinct sacrum movement patterns (Figure 3) of 13% (Pattern #1, ∆ϕ 90° in Audigie et al. 2002^15^), 25% (Pattern #2, ∆ϕ 45° in Audigie et al. 2002^15^), 38% (Pattern #3, ∆ϕ 0° in Audigie et al. 2002^15^) and 50% (Pattern #4, ∆ϕ −45° in Audigie et al. 2002^15^) was used to cover the cornerstones/boundaries of the whole pattern spectrum. Note that the pattern for a phase shift of, for example, 75% would be identical to the pattern for a phase shift of 25%, just corresponding to the opposite limb being lame.^15^ The final movement pattern was scaled to a pre‐defined movement range based on lameness severity, where the movement range was allowed to increase through a factor applied to signal component A1 (A1 factor) by 25% from 0.08 m (sound horse, A1 factor 0) to 0.1 m (moderately lame horse, A1 factor 1.5), approximating literature findings.^11^ In the model, a stride started with an early stance of the left hindlimb (LH).^18^ The supplementary video (Video S1) shows animations illustrating pattern #1 to #3; note these animations do not equate to the model but are for visualisation purposes only.

### 2.3 Pelvic roll perturbations

Possible pelvic roll adaptations by lame horses (Figure 2) were mimicked using a number of perturbations to the average pelvic rotation pattern:

- A 2° (similar to experimental observations^19^) and 5° (to test a more pronounced scenario) pelvic tilt offset to the right and left were applied, being added to the original roll signal and reflecting symmetrical rotation around a pelvis held tilted towards the left or right hindlimb.
- Asymmetry in the roll pattern was generated by increasing rotation towards the left or right hindlimb, where the amount of roll asymmetry was proportional to the lameness severity. This method mimicked descriptions indicating that horses may use asymmetrical pelvic rotation as a compensatory/biomechanical mechanism to help offload the lame limb.^11^ The respective section within the pelvic roll trajectory (increment m to n) was magnified for increased roll towards the right hindlimb (m = 1 to n = 32) and towards the left hindlimb (m = 50 to n = 82). Two different levels of asymmetry were created based on the A1 factor for a given simulation:

$$\text{Large}\;{\text{Roll}}_{\text{amp,n}:m}={\text{Roll}}_{n:m}+\left({\text{Roll}}_{n:m}\cdot \left(A1\;\text{factor}\cdot 2\right)\right)\;\text{and}$$

$$\text{Small}\;{\text{Roll}}_{\text{amp},n:m}={\text{Roll}}_{n:m}+\left({\text{Roll}}_{n:m}\cdot A1\;\text{factor}\right).$$

The magnitude of pelvic rotation (i.e. horses displaying little or lots of pelvic roll) had no impact on the predicted tubera coxae motion asymmetry and model outcomes based on prior simulations and was, therefore, not explored as a condition variable.

### 2.4 Data analysis

Outcomes (lameness/asymmetry metrics based on the positional data) for each of the five comparative tubera coxarum‐based visual assessment methods (Figure 1) were calculated for a vertical sacral motion asymmetry (MAS) ranging from 0% (perfect symmetry, sound) to 60% (moderate lameness) in 1% increments. Overall motion asymmetry of the sacrum was used as the “ground truth,” defined as representing “the reference values used as a standard for comparison purposes.”^20^ This means that the lameness classification based on the experimental data of sacrum asymmetry imposed on the model defined the “true” lameness state and severity of a given simulated horse against which the visual assessment methods relating to tuber coxae motion asymmetry were compared. This was repeated for all four sacrum movement patterns (Figure 3). Simulations of tubera coxarum movement were then repeated by introducing the perturbations to the pelvic roll pattern (Figure 2) described above, altering (a) the offset in pelvic roll (pelvis tilted by ±2° and ±5°) and (b) the asymmetry in the pelvic roll pattern (small and large asymmetry). This resulted in the simulation of 960 distinct conditions – 60 lameness severities, four movement patterns and four pelvic roll adaptations.

Graphical outcomes were produced to determine the robustness of the five visual lameness assessment methods in being able to (a) detect lameness and (b) attribute lameness to the correct limb across all simulated conditions. Robustness is here defined as a given assessment method not being susceptible to classifying a horse incorrectly based on the simulated patterns and pelvic roll perturbations.

## 3 RESULTS

**FIGURE 4:**
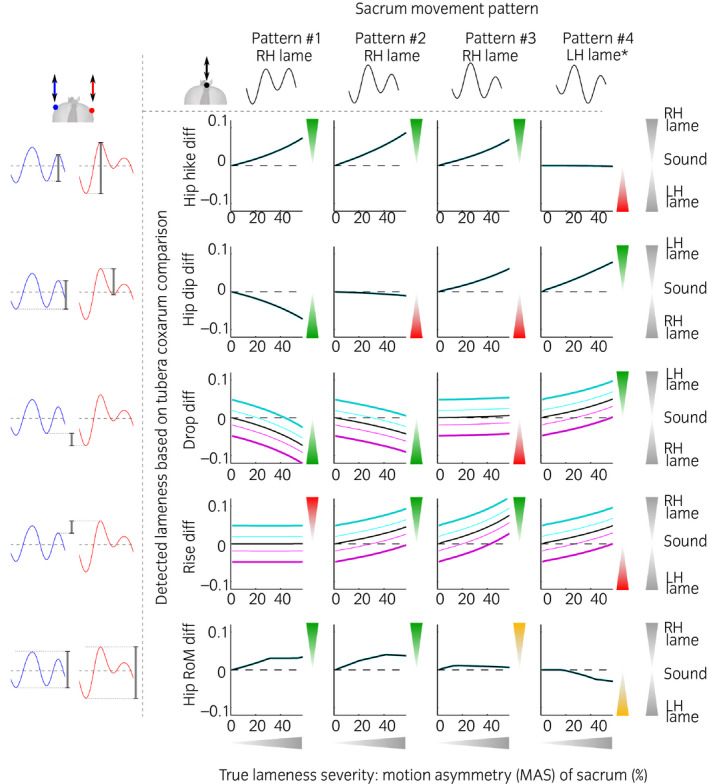
Effect of an offset in pelvic roll (pelvis held tilted) on the ability to identify lameness for the five comparative tubera coxarum‐based visual hindlimb lameness assessment methods. The pelvis is held tilted to the left (cyan, thin light: 2°, thick dark: 5°) and to the right (magenta, thin light: 2°, thick dark: 5°) compared to symmetrical pelvic roll (black). Results are shown across four different sacrum movement patterns. Lameness severities range from sound (0% sacrum motion asymmetry (MAS)) to moderately lame (60% sacrum motion asymmetry). Triangles point in the direction in which outcomes should respond. Green triangles indicate that the visual assessment would be correct for a given assessment method in horses with unaltered hip rotation, red triangles indicate that visual assessment would not be correct and orange triangles indicate that there is an issue with the visual assessment method, which may be ambiguous or disproportional. Visible lameness severity should increase proportionally to the thickness of the triangles on the secondary *y*‐axis and the values for MAS (motion asymmetry of the sacrum) on the x‐axis. As an illustrated example, the reader can see from the graphs that when using the Drop_diff assessment method (3rd row from the top) in a horse presenting with right hind lameness and a pelvis held tilted to the left (cyan), he/she will mistake a sound horse for lame (Drop_diff > 0), a mildly lame horse for sound (Drop_diff = 0) and only correctly classify a horse with moderate lameness (Drop_diff < 0), albeit perceiving lameness as less pronounced than it actually is. Please note that the sign (positive or negative) may indicate the left or right limb being lame depending on the assessment method as illustrated on the right. This is due to all assessment methods being calculated by consistently subtracting values of the left tuber coxae from the right tuber coxae. *This pattern is uncommon in practice and it remains unclear how to interpret it

**FIGURE 5:**
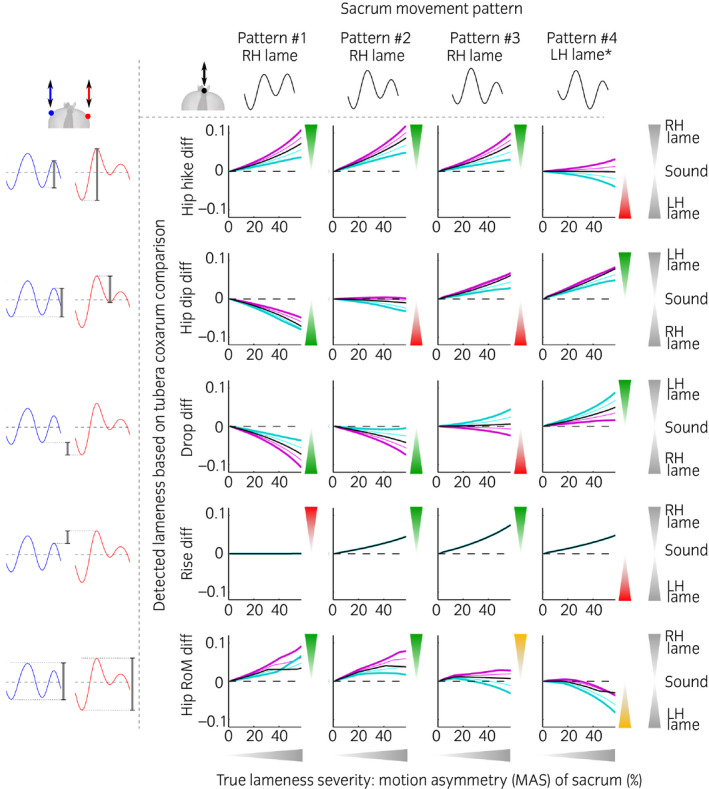
Effect of asymmetrical pelvic roll on the ability to identify lameness for the five comparative tubera coxarum‐based visual hindlimb lameness assessment methods. Asymmetrical pelvic roll is exacerbated towards the left (cyan, thin light: small amount of roll asymmetry, thick dark: a large amount of roll asymmetry) or right (magenta, thin light: small amount of roll asymmetry, thick dark: a large amount of roll asymmetry) compared to symmetrical pelvic roll (black). Please refer to Figure 4 for further details

The simulated responses of the five tubera coxarum‐based visual assessment methods to different lameness adaptation patterns are illustrated in Figures 4 and 5, with sacrum‐based motion asymmetry as the ground truth against which responses are plotted. These figures allow the exploration of potential pitfalls associated with the five specific tubera coxarum‐based visual assessment methods. Table 2 summarises all five assessment methods with respect to lameness classification and susceptibility to bias from changes in pelvic rotation. Tubera coxarum‐based lameness assessment was highly sensitive to all the unique lameness patterns and changes in pelvic rotation which a lame horse may adopt. None of the five visual lameness assessment methods was 100% robust across all conditions tested (Figures 4 and 5, Table 2). For everyday clinical practice, comparing the upward movement amplitude of the RTC before right hind foot contact and of the LTC before left hind foot contact (Hip_hike_diff) would be the most robust single tubera coxarum‐based visual assessment method.

**TABLE 2:** Performance of the five visual assessment methods for the detection and classification of hindlimb lameness based on tubera coxarum assessment

	General suitability of each visual assessment method for lameness assessment		Susceptibility of each visual assessment method to changes in pelvic rotation away from a symmetrical roll	
	Detects lameness	Determines the correct limb	Biased by roll offset (pelvis held tilted)	Biased by asymmetrical pelvic roll
Hip_hike_Diff	Pattern #1 Pattern #2 Pattern #3	Pattern #1 Pattern #2 Pattern #3	None	All patterns
Hip_dip_diff	Pattern #1 Pattern #3 Pattern #4	Pattern #1 Pattern #4	None	All patterns
Drop_diff	Pattern #1 Pattern #2 Pattern #4	Pattern #1 Pattern #2 Pattern #4	All patterns	All patterns
Rise_diff	Pattern #2 Pattern #3 Pattern #4	Pattern #2 Pattern #3	All patterns	None
Hip_RoM_diff	Pattern #1 (mild)^a^ Pattern #2 (mild)^a^ (Pattern #3)^b^ (Pattern #4)^b^	Pattern #1 Pattern #2 (Pattern #3)^b^ (Pattern #4)^b^	None	All patterns

Note

The ability of the five tubera coxarum‐based lameness assessment methods to correctly classify a horse as lame across the four movement patterns as well as their susceptibility to error based on changes in pelvic rotation. For continuous data, please refer to Figures 4 and 5.

^a^ For Hip_RoM_diff, observable severity of lameness plateaus after being proportional to the actual motion asymmetry for mild lameness.

^b^ For patterns #3 and #4, the behaviour of Hip_RoM_diff is ambiguous and disproportional, although it trends towards correct assessment.

## 4 DISCUSSION

In the current study, we show how robust five visual hindlimb lameness assessment methods based on the comparison of left and right tuber coxae movement are to variation in movement patterns and pelvic rotation. Tubera coxarum‐based assessment outcomes were compared against sacrum‐based motion asymmetry as the ground truth. Results showed that none of the five visual assessment methods tested can be expected to be 100% robust across all possible scenarios encountered by equine clinicians in everyday clinical practice. Hip_hike_diff, the difference between the upward movement amplitude of the RTC *before* RH foot contact and of the LTC *before* LH foot contact, was the most robust assessment method across conditions tested: across three of the four movement patterns and all pelvic rotation perturbations, it consistently indicated (a) the presence of lameness and (b) the correct limb. The only exception for Hip_hike_diff leading to a correct classification would be horses presenting with movement pattern #4, in which case this assessment method would not identify lameness at all. Pattern #4, however, may occur rather infrequently in practice (see below).^15^, ^17^

The high robustness of Hip_hike_diff agrees with findings from the kinematics literature, where the tuber coxae of the lame limb consistently shows an increased upward movement amplitude *before* foot contact of the lame limb.^9^, ^11^, ^21^ It is important to note that this assessment method is closely related to the assessment of successive upward movement amplitudes of the sacrum.^13^ Both approaches should provide identical results with regard to lameness classification: the only difference between sacrum‐based assessment and tubera coxarum‐based assessment should be the effect of pelvic rotation, resulting in tubera coxarum movement differing from that observable when examining movement of the sacrum. In comparison to the assessment of the sacrum, Hip_hike_diff may, however, amplify motion asymmetry^2^, ^13^ and could help to visually detect more subtle lameness. We found evidence for the exacerbation of motion asymmetry for simulations where horses increased pelvic roll towards the lame limb (Figure 5, magenta). The usefulness of Hip_hike_diff also agrees with the results from a study comparing objective and subjective lameness assessment for the evaluation of changes following nerve blocks, where the upward movement amplitude of the tubera coxarum, in line with Hip_hike_diff in this study, was deemed the most sensitive objective measure in light of subjective scores and measured asymmetry.^22^

The other four tubera coxarum‐based visual assessment methods investigated in this study were less robust across conditions tested. First, Hip_dip_diff, the comparison of downward movement amplitudes, was inconsistent in its ability to identify lameness. It showed variation in the limb it indicated as lame and did not identify lameness associated with pelvic movement pattern #2 at all. Hence, caution has to be taken when solely relying on this assessment method, as it might lead to either overlooking a lameness or classifying the incorrect limb lame. Hip_dip_diff, the magnitude of downward movement amplitudes, is not to be confused with Drop_diff, the difference in minimum heights to which the tubera coxarum drop. Second, Drop_diff was more robust in this study, echoing the literature: a study found that the tuber coxae of the lame limb often (but not always) dropped below the height of its counterpart.^2^ This lack of consistency can be explained, as Drop_diff does not identify lameness manifesting in pattern #3 at all. Drop_diff is also susceptible to changes in pelvic rotation resulting in inaccurate assessments. Third, Rise_diff, the difference in the maximum heights to which the tubera coxarum are elevated, was inconsistent in its ability to identify lameness. It showed variation in the limb it indicated as lame and did not identify lameness associated with pelvic movement pattern #1 at all. Similarly, it has been reported that the tuber coxae on the lame side did not consistently rise above the position of the contralateral tuber coxae.^2^ For Rise_diff, as for Drop_diff, an offset in pelvic roll had the potential to systematically indicate the incorrect limb as lame. However, it did not show susceptibility to asymmetrical pelvic rotation. This was due to the model assumption that rotation did not change during the part of the stride relevant to the Rise_diff metric. Fourth, Hip_RoM_diff, the difference between the total movement excursion of RTC and LTC throughout the whole stride, followed the behaviour of Hip_hike_diff for mild lameness only before plateauing (Figure 4), unless asymmetry in pelvic roll compensated for this plateau to some extent (Figure 5). In work using empirical data from live horses, Hip_RoM_diff was found to consistently indicate hindlimb lameness,^2^ likely due to the rather common occurrence of patterns #1 and #2 in horses with naturally occurring hindlimb lameness.^15^, ^17^ However, as lameness becomes more severe, this method based on the overall range of movement leads to a point of asymmetry reversal in the tubera coxarum on the sound side.^13^ After this point of reaching a plateau, the assessment method proved insensitive to lameness severity. This method is therefore not recommended for more marked lamenesses. Hip_R0M_diff is also susceptible to movement patterns and roll perturbations and fails to allow the veterinarian to grasp the total motion asymmetry visually when lamenesses become more pronounced. Hip_RoM_diff is not to be confused with Hip_hike_diff: Hip_RoM_diff assesses overall range of movement, Hip_hike_diff the upward movement at a particular point during the stride cycle. This should be particularly clarified during veterinary training.

In practice, the prevalence of the different sacrum movement patterns becomes a highly relevant factor to the probability of classifying a horse correctly during the hindlimb lameness assessment.^23^ However, at present, there are limited data available on how common these different movement patterns are in practice. An early study into signal decomposition^15^ found that of 13 horses with hindlimb lameness, seven (54%) clustered around pattern #2, three (23%) around pattern #3, two (15%) around pattern #4 and one (8%) around pattern #1. Similarly, a recent study classified horses into “impact”‐ and “push‐off”‐type lameness based on pelvic movement.^17^ In that study, impact lameness was determined based on what the authors termed “Diff Min” (the difference between the two minima of the pelvic movement trajectory) and push‐off lameness based on what they termed “Diff Max” (the difference between the two maxima of the pelvic movement trajectory).^17^ The study found that in 258 horses presenting with solely hindlimb lameness, 36% showed impact‐type lameness (corresponding to pattern #1), 40% showed push‐off‐type lameness (corresponding to pattern #3), 21% showed both types of lameness in the same limb (corresponding to pattern #2) and 4% showed impact in one and push off lameness in the other limb (corresponding to pattern #4). Together, these two studies suggest that in a broad general practice caseload, a relatively even distribution of hindlimb lameness patterns #1 to #3 can be expected, whilst pattern #4 might occur less frequently. For pattern #4, it also remains subject to debate on how to interpret it with regard to the limb it indicates as lame.

The present study explored various adaptations in pelvic rotation which lame horses may adopt. There is evidence for both, an asymmetrical roll pattern and an offset adopted by horses presenting with hindlimb lameness: First, there may be a general trend among lame horses to present an asymmetrical pelvic roll pattern characterised by increased rotation towards the lame limb. Work examining saddle slip in hindlimb lame horses supports this, where saddles slip towards the side of lameness.^24^ Secondly, a mild systematic offset in pelvic roll has been measured in lame horses.^19^ However, with limited data published on pelvic rotation in lame horses,^19^ future kinematic studies should investigate whether lame horses show systematic changes in pelvic rotation. Here, it is worth noting that there exist differences in the qualitative and quantitative description of pelvic roll adaptations during lameness in the literature.^11^, ^19^, ^25^, ^26^ Either way, individual horses are likely to adopt individual coping strategies for hindlimb lameness, especially if lameness is subtle. Hence, an individual horse may present with any of the predicted pelvic roll adaptations, and awareness of their influence on asymmetry perception will help avoid missing lameness or to declare the incorrect lame limb.

There are several limitations to consider when applying the results of this study to lameness examinations. First, in the absence of precise published data regarding the frequency of different lameness patterns and hip rotation adaptations in practice, this study cannot indicate the proportion of assessments that would be incorrect for a given visual assessment method. In future, the predictive value of different assessment methods could be calculated once such data becomes available. Second, this study uses simulated data classified based on a computer algorithm. Whilst this model is deeply rooted in and validated through data from real horses, there might be small deviations from this model for individual horses in a clinical setting. To examine this, measurement of tubera coxarum movement in a large number of horses presenting with a broad range of lameness would be valuable. However, this would require a lab setup with optical motion capture to measure the absolute position of the tubera coxarum in space. The alternative use of sensors, such as inertial measurement units (IMUs) that could be employed for collecting large datasets outside the lab, would only provide relative data and be unsuitable to quantify metrics related to some of the visual assessment methods under investigation. Third, this study is based on movement and assessment at the trot in a straight line. For assessment during trot on the circle on the lunge, bias in measurable vertical motion asymmetry has been demonstrated previously.^27^, ^28^, ^29^ Therefore, assessment on the lunge requires further research to establish the most robust methods for lameness classification, both visually and quantitatively/objectively. Fourth, whilst this study simulated motion asymmetry from 0% to 60%, the smaller levels of asymmetry investigated here may not be visible to the eye. Results of several studies now indicate an asymmetry detection threshold in the region of 15%^8^ (the just noticeable difference where 50% of horses displaying motion asymmetry are classified correctly as “lame”). At the same time, research has shown that more pronounced motion asymmetry of around 30% to 40% is required for even experienced assessors to detect both the presence of hindlimb lameness and classify the correct limb as lame in at least 50% of horses.^8^ A recent study used subjective scores from assessors viewing videos of horses to derive signal detection metrics for Min_diff and Max_diff in order to arrive at a cut‐off threshold for declaring horses as “objectively” lame.^30^ These thresholds were higher than previously reported, yet in absence of a baseline objective measure of lameness, it is unknown whether this result may also suggest difficulty in assessing videos. Overall, the accuracy with which veterinarians will be able to observe the different movement patterns and assessment methods described in this study remains to be studied.

Irrespective of the assessment method chosen, using tubera coxarum movement for the classification of hindlimb lameness requires the assessor to compare the left and right tuber coxae at specific stride timings, which is complex. It can, therefore, be beneficial to evaluate both, sacrum and comparative tubera coxarum movements during the hindlimb lameness evaluation: they should both indicate the presence of lameness and the identical limb as lame, as they are biomechanically linked.^13^ For instance, for 35 years, one of the authors (SA May) has used Hip_RoM_diff for subtle lameness, where sacral movement differences are small, and switched to sacral movement differences in more marked lameness, before comparing to one, or more, of the other visual assessment methods for hindlimb lameness evaluation.^2^ If there is a discrepancy between judgement arising from using different methods, this could indicate the presence of patterns #3 or #4, a pelvis held tilted, asymmetrical pelvic rotation or other factors that confound individual assessment methods.

## 5 CONCLUSIONS

This study showed that if hindlimb lameness evaluation is carried out through the comparative assessment of vertical tubera coxarum movement, no one assessment method is 100% robust. Using a single assessment method may hence lead to the incorrect classification of a horse as lame when it is sound, sound when it is lame or lame in the incorrect limb. The use of multiple visual assessment methods would, therefore, be beneficial to challenge perceptions and allow for comparison of lameness assessment outcomes. Hip_hike_diff, the difference between the upward movement amplitude of the RTC *before* RH touch down and of the LTC *before* LH touch down, would be the most robust single tubera coxarum‐based visual assessment method in clinical practice where lameness pattern #4 can be assumed to have a low prevalence.

## COMPETING INTERESTS

No competing interests have been declared.

## ETHICAL ANIMAL RESEARCH

Research ethics committee oversight not currently required by this journal: *in silica* studies.

## INFORMED CONSENT

Not applicable.

## AUTHOR CONTRIBUTIONS

Both authors contributed to study design and execution, data analysis and preparation of the manuscript.

## Supporting information

Video S1

Video S1

## Data Availability

Data sharing is not applicable to this article as no new data were created in this study.
