# Supplementary material for: Robustness of five different visual assessment methods for the evaluation of hindlimb lameness based on tubera coxarum movement in horses at the trot on a straight line
Source: Equine Vet J. 2021 Dec 13;54(6):1103–13. doi: 10.1111/evj.13531 (PMC9787951; doi:10.1111/evj.13531)
Supplement: Supplementary file 2 — Video S1 [file EVJ-54-1103-s001.docx]

**Robustez de cinco tipos diferentes de evaluación de cojera del miembro posterior en caballos, basado en el movimiento de las Tubera coxarum al trote en línea recta.**

Sandra D. Starke^1^ y Stephen A. May*^1^

^1^The Royal Veterinary College, Hawkshead Lane, North Mymms, Hatfield, Hertfordshire, AL9 7TA, Reino Unido.

*Email del autor corresponsal: [smay@rvc.ac.uk](mailto:smay@rvc.ac.uk)

**Palabras clave:** caballo,

**Titulo de pagina:** Evaluación de cojera del miembro posterior

**Resumen**

**Historial:** La evaluación de las cojeras del miembro posterior son un gran desafío en la clínica diaria. Sin directrices claras, los veterinarios usan métodos de evaluación diferentes para esta tarea, cuya robustez es desconocida.

**Objetivos:** Determinar la robustez de cinco métodos visuales de evaluación de cojera del miembro posterior basados en la comparación del movimiento entre la Tuber coxa derecha e izquierda.

**Diseño del estudio:** Modelo matemático validado de cojera en miembro posterior basado en datos experimentales obtenidos en la literatura.

**Métodos:** El movimiento vertical de la Tuber coxae izquierda (LTC) y derecha (RTC) fue simulado según la amplitud de movimiento de acuerdo a modelos de cojeras del miembro posterior presentados por caballos en clínica.

La gravedad de la cojera varió desde caballo sano a moderadamente cojo (0% a 60% de asimetría de movimiento). La situaciones de una pelvis inclinada y movimiento asimétrico pelviano fueron incluidos para reflejar posibles adaptaciones de rotación pélvica. A través de todas las condiciones, se cuantificaron los resultados de cinco métodos diferentes, visuales de evaluación basados en la comparación del movimiento de las Tubera coxarum, incluyendo excursión pélvica, caída pélvica y rango del movimiento. La robustez de cada método evaluativo se estableció comparándolos a la asimetría del movimiento general basado en el sacrum como verdad terreno.

**Resultados:** Las evaluaciones de cojeras basada en las Tubera coxarum fueron altamente sensibles a todos los modelos únicos de cojera y cambios en rotación pélvica posiblemente adoptados por un caballos cojo. Ninguno de los cinco métodos visuales de evaluación de cojeras fue 100% robusto en todas las condiciones probadas. Para la clínica diaria, comparar la amplitud del movimiento hacia arriba de la RTC antes de que el casco posterior derecho haga contacto con el suelo y de la LTC antes que el casco posterior izquierdo haga contacto (Hip_hike_diff) seria el único método visual de evaluación basado en Tubera coxarum mas robusto.

**Limitaciones principales:** Sin datos publicados sobre la frecuencia de los distintos modelos de movimiento y las adaptaciones de rotación pélvica en clínica, este estudio no puede indicar la proporción de evaluaciones que serian incorrectas para un método visual especifico de evaluación.

**Conclusiones:** Usar un solo método visual de evaluación de cojera del miembro posterior basado en las Tubera coxarum puede llevar a una decisión clínica incorrecta. Por lo tanto, usar múltiples métodos de evaluación seria beneficioso para fundamentar impresiones.
